# Rapidly progressive neovascular glaucoma following coronary artery bypass graft surgery in a patient with type 1 diabetes mellitus: a case report

**DOI:** 10.1186/s13256-018-1564-8

**Published:** 2018-02-12

**Authors:** Vasant Raman, Rosemary Lambley

**Affiliations:** Royal Eye Infirmary, Level 3, Derriford Hospital, Plymouth, PL6 8DH UK

**Keywords:** Proliferative diabetic retinopathy, Neovascular glaucoma, Pan-retinal photocoagulation, Coronary artery bypass graft, Case report

## Abstract

**Background:**

Proliferative diabetic retinopathy leading to vitreous hemorrhage, tractional retinal detachment, and neovascular glaucoma is a major cause of severe sight impairment in adults of working age worldwide. Neovascular glaucoma occurs in 2.5% of patients with proliferative diabetic retinopathy, which is difficult to treat and often leads to blindness. Onset of neovascular glaucoma with rapid progression to blindness within a few weeks of a successful coronary artery bypass graft procedure is not known with this clinical entity.

**Case presentation:**

A 34-year-old white man with type 1 diabetes mellitus presented to our hospital with severe, rapidly progressive bilateral neovascular glaucoma following coronary artery bypass graft surgery. The patient was previously treated for proliferative diabetic retinopathy with complete peripheral pan-retinal photocoagulation. Prompt and adequate treatment with antiglaucoma medication and cyclodiode laser did not halt the progression of the disease. The patient ended up with no perception of light within a few weeks of initial presentation.

**Conclusions:**

Vision loss following coronary artery bypass graft surgery is usually associated with nonarteritic anterior ischemic optic neuropathy. Rapid progression to bilateral blindness resulting from neovascular glaucoma within a few weeks of presentation following coronary artery bypass graft is not a common presentation with this clinical entity. Our patient had been treated with erythropoietin-stimulating factor for severe anemia preceding and following his coronary artery bypass graft surgery. The improvement in ocular perfusion following coronary artery bypass graft surgery, coupled with the administration of erythropoietin-stimulating factor, could have contributed to the onset and rapid progression of neovascular glaucoma. Close monitoring of patients with proliferative diabetic retinopathy undergoing coronary artery bypass graft surgery, even if adequately treated, is advised because there is increased ocular morbidity leading to blindness.

## Background

Neovascular glaucoma occurs in about 2.5% of patients with proliferative diabetic retinopathy [1] and is often difficult to treat, leading to a painful blind eye. Vision loss due to nonarteritic anterior ischemic optic neuropathy (AION) following coronary artery bypass graft (CABG) surgery is a well-known entity, but rapid progression of preexisting proliferative diabetic retinopathy leading to complete blindness as a result of neovascular glaucoma has not been reported, to our knowledge.

We report a case of a young adult with type 1 diabetes mellitus who developed severe progressive bilateral neovascular glaucoma within a few weeks after CABG surgery. He had proliferative diabetic retinopathy adequately treated with a pan-retinal photocoagulation (PRP) laser before undergoing cardiac surgery. This possibly could have been precipitated by treatment with erythropoietin for pre- and postoperative anemia. This clinical entity has not been reported in the English-language medical literature, to our knowledge.

## Case presentation

A 34-year-old white man with type 1 diabetes mellitus presented to our hospital with a complaint of a 3-week history of bilateral blurred vision and headaches with vomiting. He was a carpenter by profession and had insulin-dependent diabetes mellitus for 30 years. He had mild systemic hypertension and a raised cholesterol level. He smoked four or five cigarettes per day and drank alcohol only on social occasions. He described his blood sugar control as poor with his hemoglobin A1c level always above 10%. His medications included NovoRapid (Novo Nordisk, Bagsværd, Denmark) and insulin detemir, aspirin 75 mg, an angiotensin-converting enzyme inhibitor (ramipril 10 mg), and a statin (rosuvastatin 10 mg).

He had persistent active bilateral proliferative diabetic retinopathy despite full PRP treatment. Four weeks prior to his presentation, he had undergone CABG in an attempt to improve his left ventricular function and attain fitness for combined renal and pancreatic transplant surgery. Following treatment for renal disease-related anemia with an erythropoietin-stimulating agent, his preoperative hemoglobin level was recorded as 13.0 g/dl. He was again anemic with hemoglobin below 9.0 g/dl during the first postoperative week, with the lowest level being 7.7 g/dl on day 7 and with a minimum hematocrit of 0.24 L/L on day 4. He was treated with iron sucrose darbepoetin alfa infusions, and by day 34 his hemoglobin had improved to 10.7 g/dl.

On presentation, his general condition was fair, but he was in pain because of his eye condition. His pulse was 70 beats per minute, regular, and of good volume, and his blood pressure was 142/80 mmHg. He had no organomegaly and no evidence of peripheral vascular insufficiency. His heart and breath sounds were normal. On ocular examination, his visual acuity was barely counting fingers in the right eye and 6/60 (Snellen chart) in the left eye. An anterior segment examination revealed a hazy right cornea, bilateral neovascularization of the iris, and intraocular pressure of 70 mmHg in the right eye and 40 mmHg in the left by applanation tonometry. The patient had poor fundal view owing to vitreous hemorrhage. He was put on the maximum tolerated antiglaucoma medication, consisting of guttae brimonidine 0.2% twice daily to both eyes, guttae timolol 0.5% twice daily to both eyes, guttae latanoprost at night to both eyes, and acetazolamide tablet 250 mg orally three times daily. He underwent transscleral diode laser cycloablation while under peribulbar anesthesia for the pain and raised intraocular pressure. He had symptomatic relief of the pain, but his visual acuity declined to no perception of light in the right and left eyes.

## Discussion

We present a rare case of bilateral blindness resulting from rapidly developing rubeotic glaucoma in a man with type 1 diabetes who underwent CABG surgery. Blindness resulting from nonarteritic AION following CABG has been widely reported [2–6]. There is only one previous report of rubeotic glaucoma with blindness developing after CABG in a male with diabetes with a low postoperative hematocrit level [7]. Unlike our patient, he did not have any PRP laser treatment performed before the onset of neovascular glaucoma, and he did not receive exogenous erythropoietin.

Our patient’s development of aggressive neovascular glaucoma coincided with a period of postoperative anemia. Erythropoietin is an ischemia-induced angiogenic factor present at increased vitreous levels in patients with proliferative diabetic retinopathy [8]. A particular polymorphism of the erythropoietin promoter associated with increased erythropoietin production is associated with severe diabetic retinopathy and renal disease [9, 10]. It is possible that upregulation of endogenous erythropoietin production as a response to hemodilution following major surgery and the exogenously administered erythropoietin may have resulted in stimulation of florid iris new vessel growth. Both the retinas had been treated adequately by laser before the CABG surgery, with no further space to top up with supplemental laser. It is unlikely that there was a retina-induced drive for vascular proliferation in the absence of any viable retinal tissue. The high levels of angiogenic cytokines were probably effective in inducing vascular proliferation in the anterior segment. This may explain the florid neovascular glaucoma with bilateral blindness in our patient. The use of an anti-vascular endothelial growth factor agent as a disease-modulating agent early in the course of the disease remains speculative. Nevertheless, it should be considered as an option in patients with progressive disease.

## Conclusions

Rapid, progressive bilateral neovascular glaucoma after CABG surgery is a rare complication of adequately treated proliferative diabetic retinopathy. This could be accelerated in the presence of concomitant treatment with erythropoietin-stimulating factor for anemia. We suggest close monitoring of these patients because of the high morbidity with resultant blindness.

## Abbreviations

AION: Anterior ischemic optic neuropathy
CABG: Coronary artery bypass graft
PRP: Pan-retinal photocoagulation
